# Artesunate Inhibits the Cell Growth in Colorectal Cancer by Promoting ROS-Dependent Cell Senescence and Autophagy

**DOI:** 10.3390/cells11162472

**Published:** 2022-08-09

**Authors:** Zhiying Huang, Shu Gan, Xuerong Zhuang, Yao Chen, Linlin Lu, Ying Wang, Xiaoxiao Qi, Qian Feng, Qiuju Huang, Biaoyan Du, Rong Zhang, Zhongqiu Liu

**Affiliations:** 1 School of Pharmaceutical Sciences, Guangzhou University of Chinese Medicine, Guangzhou 510006, China; 2 The Second Clinical College, Guangzhou University of Chinese Medicine, Guangzhou 510006, China; 3 Guangdong-Hong Kong-Macau Joint Lab on Chinese Medicine and Immune Disease Research, Guangdong Provincial Hospital of Traditional Chinese Medicine, Guangzhou 510000, China; 4 School of Basic Medicine, Guangzhou University of Chinese Medicine, Guangzhou 510006, China

**Keywords:** artesunate, reactive oxygen species, cell senescence, autophagy, colorectal cancer

## Abstract

Although artesunate has been reported to be a promising candidate for colorectal cancer (CRC) treatment, the underlying mechanisms and molecular targets of artesunate are yet to be explored. Here, we report that artesunate acts as a senescence and autophagy inducer to exert its inhibitory effect on CRC in a reactive oxygen species (ROS)-dependent manner. In SW480 and HCT116 cells, artesunate treatment led to mitochondrial dysfunction, drastically promoted mitochondrial ROS generation, and consequently inhibited cell proliferation by causing cell cycle arrest at G0/G1 phase as well as subsequent p16- and p21-mediated cell senescence. Senescent cells underwent endoplasmic reticulum stress (ERS), and the unfolded protein response (UPR) was activated via IRE1α signaling, with upregulated BIP, IRE1α, phosphorylated IRE1α (p-IRE1α), CHOP, and DR5. Further experiments revealed that autophagy was induced by artesunate treatment due to oxidative stress and ER stress. In contrast, N-Acetylcysteine (NAC, an ROS scavenger) and 3-Methyladenine (3-MA, an autophagy inhibitor) restored cell viability and attenuated autophagy in artesunate-treated cells. Furthermore, cellular free Ca^2+^ levels were increased and could be repressed by NAC, 3-MA, and GSK2350168 (an IRE1α inhibitor). In vivo, artesunate administration reduced the growth of CT26 cell-derived tumors in BALB/c mice. Ki67 and cyclin D1 expression was downregulated in tumor tissue, while p16, p21, p-IRE1α, and LC3B expression was upregulated. Taken together, artesunate induces senescence and autophagy to inhibit cell proliferation in colorectal cancer by promoting excessive ROS generation.

## 1. Introduction

As reported by GLOBOCAN 2020, colorectal cancer (CRC) is the second leading cause of cancer-related death, with more than 1.9 million new cases and approximately 935,000 deaths estimated in 2020 [1]. In countries with economies in transition (e.g., Brazil, Slovakia, and China), the incidence rates of CRC are increasing rapidly due to a gradually westernized lifestyle, where the new annual incidence rates are even higher than those in some western countries [2]. Furthermore, screening programs and available medical treatment in many developing countries are unable to successfully achieve screening and intervention of colorectal cancer as early as possible [1]. Additionally, once patients with colorectal cancer acquire resistance to a scheduled clinical therapy, there are fairly few alternative strategies for these patients, which are detailed in the National Comprehensive Cancer Network (NCCN) guidelines [3].

Increasing studies have shown that artemisinin and its derivatives, the well-known essential anti-malarial drugs, possess diverse pharmacological activities against various diseases, including diabetes [4], autoimmune diseases [5], and cancer [6]. Recently, artesunate has entered clinical trials for cancer treatment, which suggests that artesunate is a promising drug that could be repurposed for cancer therapy. According to the study by Sanjeev Krishnea and his colleagues, artesunate reduced recurrence in patients with II/III colorectal cancer without any obvious adverse effects [7]. Several phase I/II clinic trials on the artesunate treatment against colorectal cancer are being performed. However, the underlying mechanisms and molecular targets of artesunate against colorectal cancer are still unclear.

It has already been proven that the Fe^2+^-dependent cleavage of peroxo bridges in artemisinin and its derivatives could promote excessive ROS production, causing mitochondrial dysfunction and DNA damage in plasmodium [8]. As important second messengers, ROS are indispensable in cellular signal transduction [9]. Nevertheless, excessive ROS, which is mainly induced by pathological and pharmacological stimuli, usually causes various biochemical outcomes, such as mitochondrial dysfunction, ATP reduction, lipid peroxidation, and DNA double-strand breaks, leading to repressed cell growth or increased cell death [10]. Hence, as a pathogen, pathological product, and therapeutic byproduct, ROS are proposed to be targets or an alternative strategy for the treatment of various diseases [11]. For cancer therapy, many chemical and radiation strategies are reported to function, to some extent, by inducing excessive ROS and subsequent cell death [12]. Artemisinin and its derivatives are reported to induce ROS-dependent cell death in some cancer cell lines [13,14], whereas some studies have demonstrated that artemisinin and its derivatives prevent oxidative damage in normal cells [15].

Drug repositioning, namely, exploring new indications and activities of approved drugs or yet investigated agents, is now an attractive strategy for drug discovery and development. With elucidated pharmacokinetic data and toxicological results, drug repositioning may exhibit a lower failure risk and less cost than de novo drug design, but have a higher success rate in a relatively shorter study span [16]. Artemisinin and its derivatives are ideal candidates for drug repositioning due to their disclosed extensive activities. The inhibitory effect of artesunate, a common derivative of artemisinin, on colorectal cancer has been observed clinically. We performed the present laboratory study to put insight into the underlying mechanisms for artesunate against colorectal cancer.

## 2. Materials and Methods

### 2.1. Cell Lines, Drugs and Antibodies

Colorectal cancer cell lines, SW480 and HCT116, were obtained from Chinese Academy of Science Cell Bank (Shanghai, China). CT26 cell line was purchased from CellCook Inc. (Guangzhou, China). SW480, HCT116, and CT26 were maintained in IMDM (Life Technologies, Carlsbad, CA, USA), mccoy’s 5A (HyClone, Logan, Utah, USA), and RPMI1640 (Life Technologies, Carlsbad, CA, USA), respectively, which were supplemented with 10% (*v*/*v*) fetal bovine serum (FBS, Gibico, Waltham, MA, USA). All cells were cultured at 37 °C in a humidified atmosphere containing 5% CO_2_.

Artesunate and Z-VAD-FMK were purchased from Meilunbio Inc. (Dalian, China). Necrosulfonamide, N-Acetyl Cysteine (NAC), Sodium 4-phenylbutyrate (4-PBA), and GSK2850163 were purchased from MedChemExpress LLC. (Shanghai, China). 3-Methyladenine (3-MA) was purchased from Selleck (Beijing, China). Carbonyl Cyanide m-Chlorophenylhydrazone (CCCP) was purchased from Beyotime Biotechnology (Shanghai, China). CDK2, CDK4, CDK6, Cyclin D1, Cyclin E1, p21, Rb, phosphorylated-Rb, caspase 3, cleaved-caspase 3, PARP, cleaved-PARP, Bax, Bcl-2, BIP, PERK, IRE1α, CHOP, DR5, Beclin 1, LC3A/B, Atg3, Atg5, Atg7, and Atg12 primary antibodies for western blotting were purchased from Cell Signaling Technology Inc. (Danvers, MA, USA). p16 and phosphor-IRE1α primary antibodies for western blotting were purchased from Abcam (Cambridge, UK). p16, p21, and Cyclin D1 primary antibodies for immunohistochemistry and β-actin, XBP1S, and XBP1U primary antibodies for western blotting were purchased from Proteintech Group, Inc. (Manchester, UK). LC3B was purchased from Beyotime Biotechnology (Shanghai, China). Propidium iodide (PI), RNase, 5,6-carboxyfluorescein diacetate, and succinimidyl ester (CFSE) were purchased from Sigma-Aldrich (Burlington, MA, USA).

### 2.2. Cell Viability Assay

The Enhanced Cell Counting Kit-8 (Beyotime, Shanghai, China) was used to determine cell viability. SW480 and HCT116 were seeded in 96-well plates (2.5 × 10^3^ cells per well) and treated with artesunate alone or together with Z-VAD-FMK, necrosulfonamide, NAC, 4-PBA, GSK2850163, or 3-MA for 72 h. After treatment, WTS-8 solution was added and incubated with cells for 30 min more. Absorbance was measured at 450 nm via Varioskan LUX Multimode Microplate Reader (ThermoFisher, Waltham, MA, USA).

### 2.3. ROS Detection and Quantification

For ROS source detection by confocal microscope, SW480 and HCT116 were seeded in 35 mm-glass bottom dishes (5 × 10^3^ cells per dish) and treated with artesunate at a concentration of 4 μM for 72 h. DCFH-DA (Beyotime, Shanghai, China) and MitoSOX^TM^ Red (Invitrogen) were added to medium together and incubated at 37 °C for 15 min in the dark. The final concentrations of DCFH-DA and MitoSOX^TM^ Red were 10 μM and 5 μM, respectively. Fluorescent images were captured via Leica TCS SP8 confocal microscope (Leica, Wetzlar, Germany). Average fluorescence intensity was calculated by Image J (LOCI in University of Wisconsin, Madison, WI, USA). To quantify the level of mitochondrial ROS, cells were seeded in 6-well plates (2.5 × 10^5^ cells per well) and treated with artesunate (1, 2, 4 μM) for 72 h. Cells were harvested after being loaded with MitoSOX^TM^ Red and subjected to obtain fluorescence intensity via BD FACSAria^TM^ III (BD Biosciences, Franklin Lakes, NJ, USA). The data was analyzed with FlowJo 7.6 software (BD Biosciences, Franklin Lakes, NJ, USA).

### 2.4. Cell Apoptosis Detection

Cells were seeded in 6-well plates (2.5 × 10^5^ cells per well) and treated with artesunate (1, 2, 4 μM) for 72 h. After artesunate treatment, cells were collected and incubated with 1 μg anti-Annexin V-FITC primary antibody (MACS, Frankfurt am Main, Germany) and PI solution (50 μg/mL) for 15 min at 37 °C in the dark. The results were obtained by BD FACSAria^TM^ III (BD Biosciences, Franklin Lakes, NJ, USA) and analyzed with FlowJo 7.6 software (BD Biosciences, Franklin Lakes, NJ, USA).

### 2.5. Cell Cycle Analysis

Cells were seeded in 6-well plates (2.5 × 10^5^ cells per well). Adherent cells were cultured in FBS-free medium for 24 h and then treated with artesunate at 1, 2, 4 μM for 72 h. Subsequently, all cells were harvested and fixed with 70~75% (*v*/*v*) pre-colded ethanol overnight at 4 °C. Fixed cells were washed cold 1× phosphate buffered saline (PBS) stained with 500 μL staining buffer (2% triton X-100, 50 μg/mL PI, and 100 μg/mL RNase in 1× PBS) for 15 min at room temperature (RT) in the dark. Cell cycle distribution was determined by BD FACSAria^TM^ III (BD Biosciences, Franklin Lakes, NJ, USA) and the results were analyzed with FlowJo 7.6 software (BD Biosciences, Franklin Lakes, NJ, USA).

### 2.6. Senescence-Associated β-Galactosidase (SA-β-gal) Activity Assay

SA-β-gal activity was measured using the SA-β-gal staining kit (Beyotime, Shanghai, China). SW480 and HCT116 were seeded in 6-well plates (2.5 × 10^5^ cells per well) treated with artesunate alone or together with NAC for 72 h. After treatment, cells were fixed and then stained with the instant-prepared staining working solution overnight at 37 °C without CO_2_ following the manufacturer’s instructions. Positive-stained cells were observed and imaged with optical microscope (Leica, Wetzlar, Germany).

### 2.7. Cell Proliferation Assay

Cells were seeded in 6-well plates (2.0 × 10^5^ cells per well) and treated with aresunate for 72 h. Then, cells were incubated with CFSE at a final concentration of 5 μM at 37 °C in the dark for 30 min. Afterwards, cells were rinsed with 1×PBS three times and then cultured in CFSE-free medium (containing 10% FBS) for 6 h. The fluorescence intensity of intracellular CFSE were quantified by BD FACSAria^TM^ III (BD Biosciences, Franklin Lakes, NJ, USA).

### 2.8. Mitochondrial Membrane Potential Assay

JC-1 (BD Biosciences, Franklin Lakes, NJ, USA) was used to detect the mitochondrial membrane potential. SW480 and HCT116 were seeded in 6-well plates and exposed to artesunate at 1, 2, 4 μM for 24 h. Subsequently, JC-1 was added to incubate with cells in a CO_2_ incubator at 37 °C for 15 min. After that, cells were collected to analyze by BD FACSAria^TM^ III (BD Biosciences, Franklin Lakes, NJ, USA). The fluorescence intensity of both JC-1 monomers and JC-1 aggregates was recorded.

### 2.9. Mitophagy Detection

Mitophagy was detected using the mitophagy detection kit (Dojindo, Kumamoto, Japan). SW480 and HCT116 cells were seeded in 35 mm-glass bottom dishes (5 × 10^3^ cells per dish) and incubated with mitophagy dye at a final concentration of 100 nM at 37 °C in a CO_2_ incubator for 30 min. Later, cells were rinsed with FBS-free medium twice and exposed to artesunate or other drugs (NAC, CCCP, etc.). After treatment, lyso dye was added at a final concentration of 1 μM and incubated for 30 min under the same conditions. Images were captured via Leica TCS SP8 confocal microscope (Leica, Wetzlar, Germany).

### 2.10. Lysosomal Acidification Detection

Lysosomal acidification was determined via LysoTracker^®^ Green DND-26 (Cell Signaling Technology). SW480 and HCT116 cells were seeded in 6-well plates (2.5 × 10^5^ cells per well) and treated with artesunate at 1, 2, and 4 μM for 72 h. After treatment, LysoTracker^®^ Green DND-26 at a final concentration of 50 nM was added and incubated for 15 min at 37 °C in the dark. Afterwards, cells were collected to quantify fluorescence intensity via BD FACSAria^TM^ III (BD Biosciences, Franklin Lakes, NJ, USA). Fluorescence intensity was analyzed by FlowJo 7.6 software (BD Biosciences, Franklin Lakes, NJ, USA).

### 2.11. Plasmid Transfection and Enhanced Green Fluorescent Protein (EGFP)-LC3B Quantification

SW480 and HC116 were seeded in 6-well plates (1.5 × 10^5^ cells per well). For each well, 2.5 μg pEGFP-LC3B-C5 plasmids were transfected into cells with 5 μL Lipofectamine^TM^ 2000 Transfection Reagent (Invitrogen). Transfected cells were treated with artesunate alone or together with NAC for 72 h. When finished, cells were harvested to determine the fluorescence intensity of EGFP via BD FACSAria^TM^ III (BD Biosciences, Franklin Lakes, NJ, USA).

### 2.12. Cellular Calcium Detection

Fluo 4-AM (YEASEN, Shanghai, China) was applied to determine the level of cellular free calcium. SW480 and HCT116 were seeded in 6-well plates (2.5 × 10^5^ cells per well) and treated with artesunate at 1, 2, and 4 μM for 72 h. Once the treatment finished, cells were loaded with Fluo 4-AM at a final concentration of 1 Μm for 30 min at 37 °C in the dark and then collected to read fluorescence intensity via BD FACSAria^TM^ III (BD Biosciences, Franklin Lakes, NJ, USA). The data were analyzed and visualized by FlowJo 7.6 software (BD Biosciences, Franklin Lakes, NJ, USA).

### 2.13. Western Blotting

SW480 and HC116 were seeded in 60 mm dishes (5 × 10^5^ cells per dish) and treated for 72 h. Cells extracts were prepared with RIPA buffer (GBCBIO, Guangzhou, China) and quantified using BCA reagent (Beyotime, Shanghai, China). In total, 20 μg denaturized cell extracts were separated in sodium dodecyl sulfate-polyarcylamide gel electrophoresis (SDS-PAGE) at 90 V for 120 min and then transferred onto PVDF membrane (0.22 μM, Millipore, Burlington, MA, USA) at 300 mA for about 60~90 min. Electrophoresis and electroblotting were carried out using Mini-PROTEAN Tetra Cell (Bio-Rad, Hercules, CA, USA). The protein-loaded membranes were blocked with 5% milk (Beyotime, Shanghai, China), which was solved in 1 × tris-buffered saline (with 0.1% tween, TBST), at RT for 1 h and then incubated with primary anti-bodies (1:1000 diluted in 1% BSA) overnight at 4 °C, following the incubation with secondary antibodies (1:3000 diluted in 1% BSA) for 60 min at RT on the next day. Images of protein blots on membranes were obtained with enhanced chemiluminescence (ECL) reagent (GBCBIO, Guangzhou, China) using FluorChem E (ProteinSimple, San Jose, CA, USA). The gray values of protein blots were evaluated by Image J (LOCI in University of Wisconsin, Madison, WI, USA). Relative protein expression was normalized to β-actin.

### 2.14. CT26-Derived Implanted Tumor Model

Mice-sourced colorectal cancer cells CT26 were injected (1 × 10^5^ cell for each mouse) subcutaneously at the right axillary of balb/c mice to establish implanted tumor model. Tumor-loaded mice were gavaged with artesunate at 30 mg/kg or 60 mg/kg for 24 days. Body weights and tumor volumes were recorded every three days. Curves for body weight and tumor volume were plotted. Tumor tissues were collected from sacrificed mice and fixed in 4% paraformaldehyde (Servicebio, Wuhan, China) for further analysis. The animal experiment was approved by Animal Care and Use Committee (IACUC) in Guangzhou University of Chinese Medicine.

### 2.15. Immunohistochemistry (IHC)

SABC-POD staining kit (BOSTER, Wuhan, China) was used to perform immunohistochemical staining. For IHC staining, 4 μm-sections were prepared from paraffin-embedded tumor tissues. After dewaxing and rehydration, sections were incubated with 3% H2O2 at RT for 15 min and then boiled in 0.01 M citrate buffer (pH 6.0). Subsequently, sections were incubated with primary antibodies against Ki67, Cyclin D1, p16, p21, phosphorylated-IRE1α, and LC3B overnight at 4 °C in a humidified box and then incubated with biotin-labeled secondary antibody at RT for 1 h. Positive signal of target protein was amplified by streptavidin and biotin-labeled horseradish peroxidase in SABC-HRP kit (Beyotime, Shanghai, China). Images were captured by optical microscope (Leica, Wetzlar, Germany).

### 2.16. Statistical Analysis

Statistical analyses were carried out by SPSS 26.0. Multiple comparisons were performed via one-way ANOVA, and comparisons between the two groups were performed via Student’s *t*-test. Data were presented as mean ± standard deviation (SD). Curve charts and bar charts were plotted by GraphPad Prism 8.0 (GraphPad Software, San Diego, CA, USA). A *p* ≤ 0.05 is determined as significance. All experiments were performed at least in triplicates.

## 3. Results

### 3.1. Artesunate Inhibited Cell Viabilities Partially due to Excessive Mitochondrial ROS

**Figure 1:**
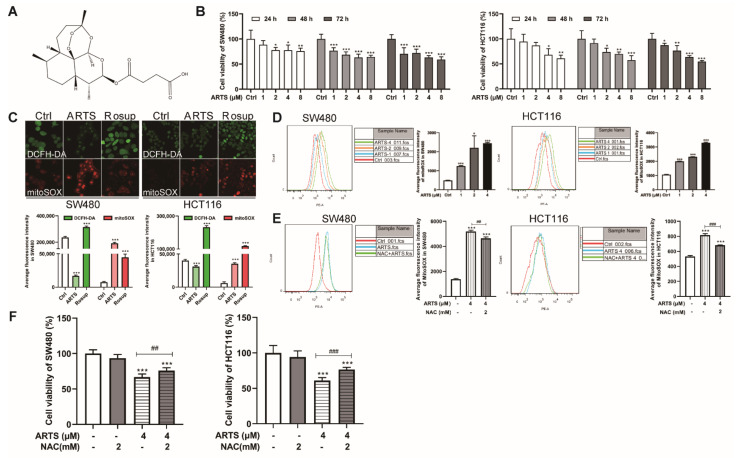
Artesunate inhibited cell viabilities partially due to excessive mitochondrial ROS. (**A**) The endoperoxide structure of artesunate. (**B**) Artesunate inhibited cell viabilities of SW480 and HCT116 dose-dependently. The colorectal cancer cell lines, SW480 and HCT116, were seeded in 96-well plates and treated with artesunate at 1, 2, 4, and 8 μM for 24, 48, 72 h. Cell viabilities were detected via CCK8 assay. (**C**) Artesunate caused mitochondrial ROS generation. Cells were seeded in glass-bottomed dishes and treated with artesunate (4 μM) for 72 h. DCFH-DA (Green) and MitoSOX^TM^ Red (Red) was used to label the total intracellular ROS and mitochondrial specific ROS respectively. Images were captured by confocal microscope (Scale bar = 25 μm). Rosup was used as a positive agent of ROS inducer. (**D**) Mitochondrial ROS was increased dose-dependently. Cells were probed with MitoSOX^TM^ Red after artesunate treatment. The fluorescence intensity of MitoSOX^TM^ Red was record by flow cytometry to indicate the mitochondrial ROS level. The ROS scavenger NAC helped to (**E**) decrease the mitochondrial ROS, and (**F**) restore cell viabilities in treated SW480 and HCT116. NAC was used at a concentration of 2 mM and added alone or together with artesunate (4 μM) for 72 h before cell viability assay and MitoSOX^TM^ Red incubation. * *p* < 0.05, ** *p* < 0.01, and *** *p* < 0.001 vs. Ctrl. ## *p* < 0.01, and ### *p* < 0.001 vs. cells treated with artesunate alone. Data were shown as mean ± SD.

The antitumor effects of artesunate on colorectal cancer cells were evaluated in two cell lines, SW480 and HCT116. The CCK-8 assay results suggested that artesunate inhibited the viability of SW480 and HCT116 cells in a dose-dependent manner (Figure 1B). When treated with artesunate at 1, 2, 4, and 8 μM for 72 h, cell viability ranged from 70.34% ± 12.35% (1 μM, *p* < 0.001) to 59.00% ± 5.61% (8 μM, *p* < 0.001) in SW480 cells and from 87.25% ± 4.09% (1 μM, *p* < 0.001) to 54.68% ± 2.04% (8 μM, *p* < 0.001) in HCT116 cells. Accordingly, artesunate at 1, 2, and 4 μM was selected for subsequent studies.

Usually, the endoperoxide function of artesunate is thought to produce ROS. Mitochondria are one of the main sources of cellular ROS [17]. As expected, artesunate mediated excessive ROS accumulation in mitochondria, as indicated by MitoSOX^TM^ Red (Figure 1C). As analyzed by flow cytometry, the fluorescence intensity of MitoSOX^TM^ Red in treated SW480 cells was approximately 1.54 ± 0.06 (1 μM, *p* < 0.001), 3.49 ± 1.23 (2 μM, *p* < 0.05), and 3.98 ± 0.09 (4 μM, *p* < 0.001) times greater than that in untreated SW480 cells (Figure 1D). Compared to untreated HCT116 cells, the fluorescence intensity of MitoSOX^TM^ Red in treated HCT116 cells was approximately 0.87 ± 0.02 (1 μM, *p* < 0.001), 1.17 ± 0.02 (2 μM, *p* < 0.001), and 2.10 ± 0.04 (4 μM, *p* < 0.001) times greater (Figure 1D).

When treated with artesunate and NAC simultaneously, the excessive ROS levels in co-treated SW480 and HCT116 cells was decreased by approximately 10.71% ± 2.20% (*p* < 0.01, Figure 1E) and 16.55% ± 0.88% (*p* < 0.001, Figure 1E), respectively. Furthermore, cell viabilities in SW480 and HCT116 cells were increased by approximately 13.88% ± 6.08% (*p* < 0.01, Figure 1F) and 25.87% ± 4.68% (*p* < 0.001, Figure 1F) due to the NAC exposure.

### 3.2. Artesunate-Induced Mild Apoptosis Did Not Help to Inhibit the Viability of SW480 and HCT116 Cells

**Figure 2:**
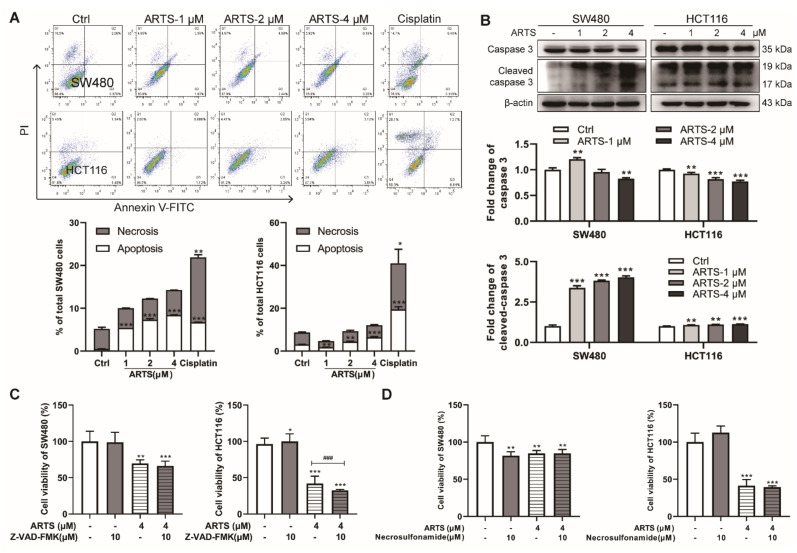
Artesunate-induced mild apoptosis could not help to inhibit cell viabilities of SW480 and HCT116. (**A**) Artesunate only induced mild apoptosis in SW480 and HCT116. Cells were seeded in 6-well plates and treated with artesunate (1, 2, and 4 μM) for 72 h. Afterwards, all cells (suspended and attached) were harvested. After incubation with anti-Annexin V-FITC primary antibody and PI solution, apoptotic cells were detected via flow cytometry. Cisplatin was used as positive drug for cell apoptosis at a concentration of 30 μM for 24 h. (**B**) Artesunate upregulated the protein level of cleaved-caspase 3 in SW480 and HCT116. Cells were seeded in 60 mm dishes and treated with artesunate (1, 2, and 4 μM) for 72 h. Then, cells were lysed with RIPA to extract total protein. The protein levels were measured by western blotting. The gray values of protein blots were evaluated by Image J. Relative protein expression was normalized to β-actin. (**C**) Z-VAD-FMK, a pan-caspase inhibitor, and (**D**) necrosulfonamide, a necrosis inhibitor, could not help to restore cell viabilities. Z-VAD-FMK and necrosulfonamide were used at a concentration of 10 μM and added alone or together with artesunate (4 μM) for 72 h. Cell viabilities were detected via CCK8 assay. * *p* < 0.05, ** *p* < 0.01, and *** *p* < 0.001 vs. Ctrl. ### *p* < 0.001 vs. cells treated with artesunate alone. Data were shown as mean ± SD.

According to the Annexin-V FITC/PI assay, less than 10% of SW480 and HCT116 cells underwent apoptosis even when treated with artesunate for 72 h (*p* < 0.05, Figure 2A). The protein level of cleaved-caspase 3, the active form of caspase 3, was induced after artesunate treatment (Figure 2B). However, we found that neither Z-VAD-FMK (a pancaspase inhibitor) nor necrosulfonamide (a necrosis inhibitor) helped to restore the viability of SW480 and HCT116 cells (Figure 2C,D, respectively). These results indicated that artesunate-induced cell apoptosis did not help to inhibit the viability of SW480 and HCT116 cells.

### 3.3. Artesunate Induced Cell Senescence ROS-Dependently

**Figure 3:**
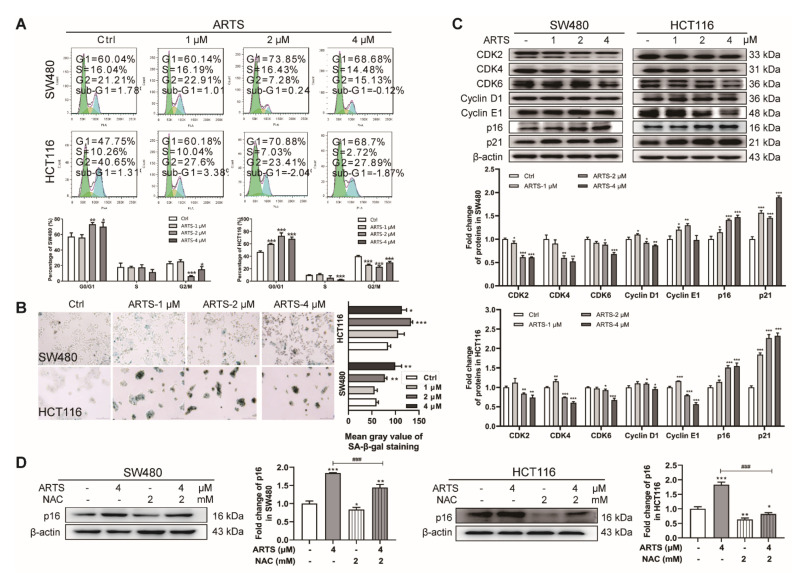
Artesunate caused excessive mitochondrial ROS to induce cell senescence and inhibit cell proliferation. (**A**) Artesunate arrested cell cycle at G0/G1 phase in SW480 and HCT116. Cells were seeded in 6-well plates. Once attached, cells were maintained in FBS-free medium for about 16~24 h. Afterwards, cell were treated with artesunate (1, 2, and 4 μM in medium containing 10% FBS) for 72 h and then harvested for PI staining to analyze cell cycle by flow cytometry. (**B**) Artesunate induced cell senescence in SW480 and HCT116. Cells were seeded in 6-well plates and treated with artesunate (1, 2, and 4 μM) for 72 h. Cell senescence was represented via SA-β-gal activity, which was assayed using a SA-β-gal staining kit after artesunate treatment. (**C**) Artesunate treatment downregulated the protein levels of CDK 2/4/6 and upregulated the protein levels of CDKIs, p16, and p21 in SW480 and HCT116. Cells were seeded in 60 mm dishes and treated with artesunate (1, 2, and 4 μM) for 72 h. Then, cells were lysed with RIPA to extract total protein. The protein levels were measured by western blotting. The gray values of protein blots were evaluated by Image J. Relative protein expression was normalized to β-actin. (**D**) NAC attenuated the effect of artesunate on protein expression of p16 in SW480 and HCT116. NAC was used at a concentration of 2 mM and added alone or together with artesunate (4 μM) for 72 h. * *p* < 0.05, ** *p* < 0.01, and *** *p* < 0.001 vs. Ctrl. ### *p* < 0.001 vs. cells treated with artesunate alone. Data were shown as mean ± SD.

As shown in Figure 3A, when treated with artesunate for 72 h, the percentage of SW480 cells at G0/G1 phase was increased from 57.38% ± 3.98% (control group) to 73.21% ± 1.86% (2 μM, *p* < 0.01), 70.19% ± 4.57% (4 μM, *p* < 0.05), while the percentage of HCT116 cells increased from 47.02% ± 31.44% (control group) to 59.90% ± 0.76% (1 μM, *p* < 0.001), 72.85% ± 3.89% (2 μM, *p* < 0.001), 68.16% ± 2.22% (4 μM, *p* < 0.001).

Senescent cells are characterized by irreversible cell cycle arrest [18]. To verify whether artesunate-treated cells underwent cell senescence, the activity of SA-β-gal, a lysosomal enzyme acting as a marker for the increased lysosomal content in senescent cells [19], was measured using x-gal as the substrate. In artesunate-treated SW480 and HCT116 cells, the number of SA-β-gal-positive cells was increased, and the x-gal-sourced blue product catalyzed by SA-β-gal in each senescent cell accumulated in a dose-dependent manner (Figure 3B). Furthermore, an enlarged and flattened morphology was observed in artesunate-induced senescent cells (Figure S1A).

Senescent cells exhibit little proliferative activity. Accordingly, we performed a CFSE-based assay to monitor the inhibitory effect of artesunate on cell proliferation. CFSE tracks cell proliferation via dye dilution during cell division [20]. As shown in Figure S1B, due to inhibited cell division, CFSE could not be diluted. The mean fluorescence density of CFSE in artesunate treated cells was remarkably higher than untreated cells.

The protein levels of CDK2/4/6, the main regulators of the G1/S checkpoints in the cell cycle, were reduced in both SW480 and HCT116 cells (Figure 3C), confirming that artesunate caused cell cycle arrest at the G1 phase in SW480 and HCT116 cells. Endogenic inhibitors of CDKs, p16 and p21, the reported main drivers of cell cycle arrest in senescence [21,22], were upregulated by artesunate (Figure 3C). As reported, in senescent cells, p16 and p21 repress CDK2/4/6 and cyclin D/E activity, leading to Rb hypophosphorylation and resulted in downregulated kinase activity of Rb [23]. Consistently, the phosphorylation level of Rb (p-Rb) was reduced after artesunate treatment (Figure S1C). Notably, the total protein level of Rb was also downregulated in treated SW480 cells (Figure S1C).

When cells were co-treated with artesunate and NAC, the number of SA-β-gal-positive cells and the accumulation of the x-gal-sourced blue product were less than that in cells treated with artesunate only (Figure S1D). Additionally, the protein level of p16 in artesunate-treated cells was markedly downregulated by NAC co-treatment (*p* < 0.001, Figure 3D). These results suggested that artesunate induced cell senescence and inhibited cell proliferation, to some extent, partially in a ROS-dependent manner. Comparatively, NAC exhibited a greater inhibitory effect on p16 protein expression in HCT116.

### 3.4. Artesunate Promoted Mitochondrial Depolarization and Mitophagy

**Figure 4:**
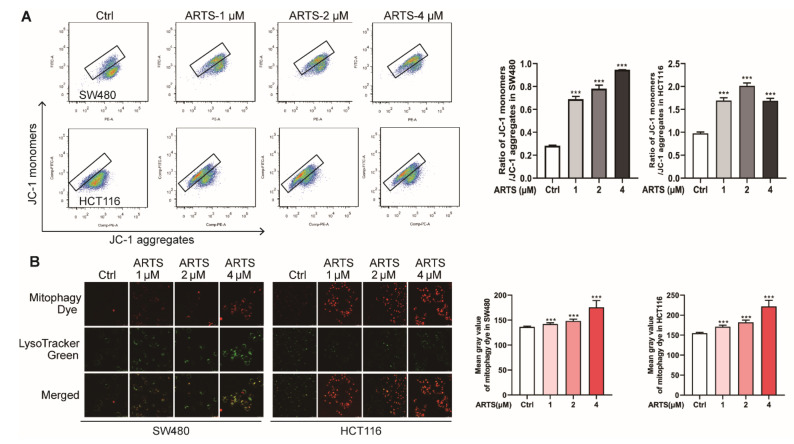
Artesunate promoted mitochondria depolarization and resulted in mitophagy. (**A**) Artesunate caused mitochondria depolarization. Cells were seeded in 6-well plates and treated with artesunate (1, 2, 4 μM) for 24 h and then incubated with JC-1. The average intensity of green fluorescence from cytoplasmic JC-1 monomers and the red fluorescence from JC-1 aggregates was recorded by flow cytometry. The ratio between JC-1 monomers and JC-1 aggregates represented mitochondria depolarization and the damaged mitochondrial membrane permeability. (**B**) Artesunate induced mitophagy in SW480 and HCT116 dose-dependently. Cells were seeded in glass-bottomed dishes. Cells were loaded with mitophagy dye before artesunate treatment. After being treated with artesunate (1, 2, and 4 μM) for 72 h, images were captured immediately by confocal microscope (Scale bar = 25 μm). The intensity of red fluorescence was calculated by Image J. *** *p* < 0.001 vs. Ctrl. Data were shown as mean ± SD.

As shown in Figure 4A, compared to the untreated SW480 cells, the ratio between the green fluorescence from cytoplasmic JC-1 monomers and red fluorescence from JC-1 aggregates in mitochondria was increased from 0.28 ± 0.01 (the control group) to 0.69 ± 0.02 (1 μM, *p* < 0.001), 0.78 ± 0.03 (2 μM, *p* < 0.001), and 0.95 ± 0.01 (4 μM, *p* < 0.001), while the ratio in HCT116 cells was increased from 0.98 ± 0.03 to 1.70 ± 0.05 (1 μM, *p* < 0.001), 2.03 ± 0.04 (2 μM, *p* < 0.001), and 1.70 ± 0.04 (4 μM, *p* < 0.001). These results indicated that artesunate caused mitochondria damage and reduced mitochondrial membrane permeability, leading to mitochondrial depolarization and dysfunction.

Mitochondria dysfunction is proven to be a main modulator of cell senescence [24]. Mitochondria damage causes cellular ROS generation and activates to mitophagy for degradation [25]. As illustrated in Figure 4B, when treated with artesunate for 72 h, the mitophagy dye accumulated in cells in a dose-dependent manner (*p* < 0.001). The intensity of mitophagy dye in SW480 was increased from 136.35 ± 1.54 (the control group) to 142.05 ± 2.47 (1 μM, *p* < 0.001), 148.40 ± 3.47 (2 μM, *p* < 0.001), and 175.63 ± 13.64 (4 μM, *p* < 0.001), while that in HCT116 was increased from 154.90 ± 2.08 (the control group) to 171.00 ± 4.18 (1 μM, *p* < 0.001), 182.04 ± 5.57 (2 μM, *p* < 0.001), and 221.81 ± 15.39 (4 μM, *p* < 0.001).

### 3.5. Artesunate Induced Autophagy to Inhibit Cell Proliferation Mainly due to the Excessive ROS Generation

Autophagy is the mechanism by which damaged cellular components are degraded to prevent cells from experiencing various stresses [26]. As demonstrated by flow cytometry (Figure S3A), the intensity of LysoTracker^®^ Green DND-26 in SW480 cells was increased by 9.74% ± 0.46% (2 μM, *p* < 0.001) and 12.92% ± 1.41% (4 μM, *p* < 0.001), while that in HCT116 cells was increased by 58.37% ± 0.49% (1 μM, *p* < 0.001), 88.38% ± 2.33% (2 μM, *p* < 0.001), and 111.93% ± 10.41% (4 μM, *p* < 0.001). The results showed that artesunate promoted lysosomal acidification, showing the potential to activate autophagy.

**Figure 5:**
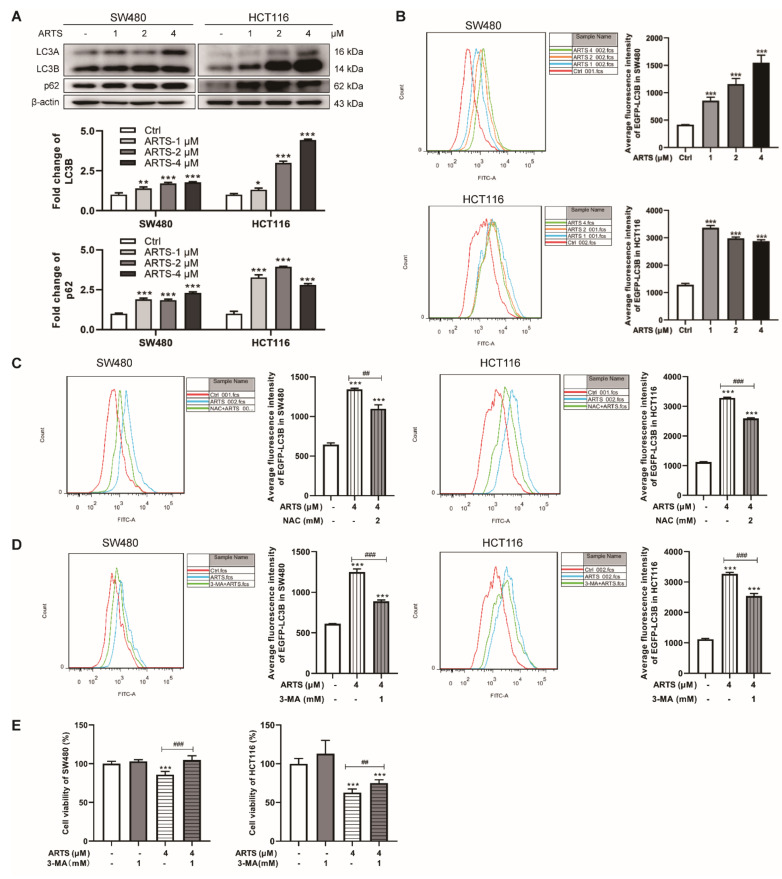
Artesunate caused autophagy to inhibit cell proliferation ROS-dependently. (**A**) Artesunate upregulated the expression levels of LC3B and p62. Cells were seeded in 60 mm dishes and treated with artesuante (1, 2, 4 μM) for 72 h. Then, cells were lysed with RIPA to extract total protein. The protein levels were measured by western blotting. The gray values of protein blots were evaluated by Image J. Relative protein expression was normalized to β-actin. (**B**) Artesunate induced the expression of exogenous LC3B. SW480 and HCT116 were transfected with pEGFP-LC3B-C1 plasmid and then treated with artesunate (1, 2, and 4 μM) for 72 h. The fluorescence intensity of EGFP, which was analyzed by flow cytometry, represented the expression level of exogenous LC3B. (**C**) NAC helped to reduce the fluorescence intensity of EGFP. 3-MA was used at a concentration of 1 μM and added alone or together with artesunate (4 μM) for 72 h. 3-MA, an autophagy inhibitor, helped to (**D**) reduced the fluorescence intensity of EGFP and (**E**) restore cell viabilities of SW480 and HCT116. * *p* < 0.05, ** *p* < 0.01, and *** *p* < 0.001 vs. Ctrl. ## *p* < 0.01, and ### *p* < 0.001 vs. cells treated with artesunate alone. Data were shown as mean ± SD.

LC3B, the typical marker for autophagosomes, was induced in artesunate-treated cells does-dependently (Figure 5A). Simultaneously, p62, an autophagy receptor that facilitates the formation of autophagosomes [27], was also upregulated (Figure 5A). Additionally, artesunate treatment increased the expression levels of several autophagy-related proteins (Atgs), including Beclin1, Atg3, Atg5, Atg7, and Atg12 (Figure S3D).

The effect of artesunate on the expression of LC3B was further confirmed by a pEGFP-LC3B-C5 plasmid. The expression level of exogenous LC3B, which fused with EGFP, was quantified by the fluorescence intensity of EGFP. As illustrated in Figure 5B, the fluorescence intensity of EGFP-LC3B in transfected and treated SW480 cells was approximately 2.10 ± 0.16 (1 μM, *p* < 0.001), 2.77 ± 0.25 (2 μM, *p* < 0.05), and 3.71 ± 0.22 (4 μM, *p* < 0.001) times greater than that in transfected cells without treatment, while in HCT116 cells, it was approximately 2.62 ± 0.07 (1 μM, *p* < 0.001), 2.32 ± 0.04 (2 μM, *p* < 0.001), and 2.24 ± 0.04 (4 μM, *p* < 0.001) times greater.

Notably, NAC exposure led to a decrease of EGFP-LC3B levels in treated SW480 and HCT116 cells. As monitored by flow cytometry, the fluorescence intensity of EGFP-LC3B in transfected SW480 and HCT116 was decreased by about 18.25% ± 3.63% (*p* < 0.01, Figure 5C) and 21.10% ± 0.88% (*p* < 0.001, Figure 5C) due to NAC exposure, respectively. Additionally, after NAC treatment, the fluorescence intensity of LysoTracker^®^ Green DND-26 in treated SW480 and HCT116 cells was decreased by approximately 12.68% ± 2.60% (*p* < 0.001, Figure S3B) and 49.65% ± 0.56% (*p* < 0.001, Figure S3B), respectively. These results emphasized that artesunate induces autophagy, including mitophagy, mainly due to excessive ROS.

Compared to that in cells treated with artesunate only, 3-MA (an autophagy inhibitor) exposure decreased the fluorescence intensity of EGFP-LC3B in transfected SW480 and HCT116 by approximately 28.83% ± 1.14% (*p* < 0.001, Figure 5D) and 22.29% ± 2.52% (*p* < 0.001, Figure 5D), respectively. Meanwhile, the viability of SW480 and HCT116 cells, which were co-treated with artesunate and 3-MA, was increased by approximately 21.99% ± 6.53% (*p* < 0.001, Figure 5E) and 22.43% ± 10.90% (*p* < 0.001, Figure 5E), respectively. In addition, when 3-MA was added, the fluorescence intensity of LysoTracker^®^ Green DND-26 in artesunate-treated SW480 and HCT116 cells was decreased by approximately 52.83% ± 2.03% (*p* < 0.001, Figure S3C), 14.14% ± 2.24% (*p* < 0.01, Figure S3C), respectively. These results indicated artesunate inhibited cell viability partially depending on the activation of autophagy.

### 3.6. Artesunate Induced Endoplasmic Reticulum Stress (ER Stress) and Activated the Unfolded Protein Response (UPR) via IRE1α Signaling

**Figure 6:**
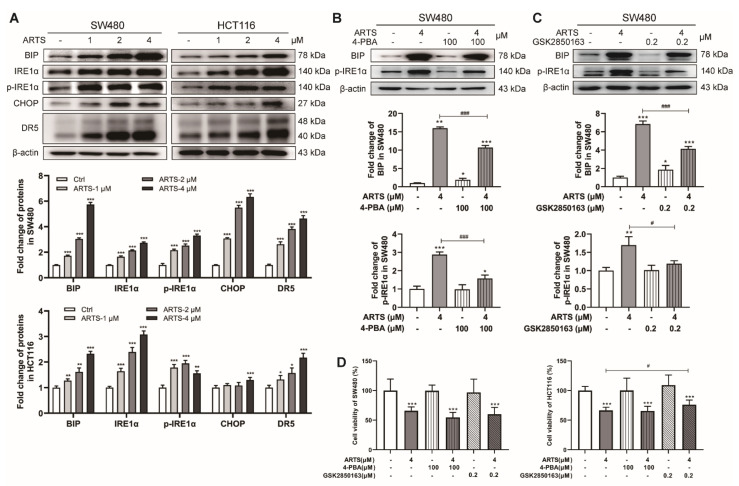
Artesunate induced ER stress and activated UPR via IRE1α signaling. (**A**) Artesunate induced the expression of ER stress sensor BIP and activated the IRE1α branch of UPR pathways. The protein levels of BIP, IRE1α, phosphorylated-IRE1α (p-IRE1α), CHOP, and DR5 were detected by western blotting. (**B**) The ER stress inhibitor 4-PBA and (**C**) IRE1α inhibitor GSK2350168 helped to reduce the protein levels of BIP and p-IRE1α. (**D**) GSK2350168 helped to restore cell viabilities in HCT116. 4-PBA was used at a concentration of 100 μM and added alone or together with artesunate (4 μM) for 72 h. GSK2350168 was used at a concentration of 0.2 μM and added alone or together with artesunate (4 μM) for 72 h. * *p* < 0.05, ** *p* < 0.01, and *** *p* < 0.001 vs. Ctrl. # *p* < 0.05, ### *p* < 0.001 vs. cells treated with artesunate alone. Data were shown as mean ± SD.

Oxidative stress may cause endoplasmic reticulum dysfunction, leading to ER stress and resulting in the UPR [28]. In addition, the senescence-related secretory phenotype of senescent cells promotes the synthesis activity of secreted proteins in endoplasmic reticulum, which may also lead to ER stress and UPR [29]. BIP, a sensor for ER stress with good affinity for unfolded and misfolded proteins [30], was markedly induced by artesunate (*p* < 0.001, Figure 6A). IRE1α, which is activated through autophosphorylation when dissociated from BIP, was remarkably upregulated and hyperphosphorylated in artesunate-treated cells (*p* < 0.001, Figure 6A). The spliced form of XBPI (XBP1S), a downstream target of IRE1α, was increased, while the unspliced form of XBPI (XBP1U) was decreased (Figure S4A). Notably, CHOP and its target DR5, which are proven to be effectors that regulate cell apoptosis in the UPR, were induced by artesunate (*p* < 0.001, Figure 6A). Nevertheless, the expression levels of PERK showed no obvious change (Figure S4A).

4-PBA (an ER stress inhibitor) and GSK2850163 (an IRE1α inhibitor) were applied to verify the effects of artesunate on ER stress and IRE1α-mediated UPR. 4-PBA and GSK2850163 reduced the levels of BIP and phosphorylated IRE1α in SW480 and HCT116 cells (Figure 6B,C and Figure S4B,C). The levels of XBP1S and DR5 were also reduced by GSK2850163 and 4-PBA (Figure S4D,E). However, the results of the CCK-8 assay revealed that GSK2850163 could only restore the viability of HCT116 cells by approximately 14.33% ± 12.09% (*p* < 0.05, Figure 6D).

Moreover, we used 4-PBA and GSK2850163 to determine whether activated autophagy in an ER stress-dependent manner. As monitored by flow cytometry, GSK2850163 exposure resulted in a decrease of the fluorescence intensity of EGFP-LC3B by approximately 15.45% ± 4.70% (*p* < 0.01, Figure S4F) in SW480 cells and 21.69% ± 2.33% in HCT116 cells (*p* < 0.001, Figure S4F). When cells were exposed to 4-PBA, the fluorescence intensity of EGFP-LC3B was decreased by approximately 7.05% ± 5.02% (*p*>0.05, Figure S4F) in SW480 cells and 11.52% ± 0.48% in HCT116 cells (*p* < 0.001, Figure S4F). The results suggested that activated ER stress and UPR, especially the activation of IRE1α signaling, may play a role in promoting autophagy in artesunate-treated CRC cells. However, 4-PBA and GSK2850163 decreased the fluorescence intensity of LysoTracker^®^ Green DND-26 in HCT116 cells by only approximately 9.71% ± 1.49% (*p* < 0.05, Figure S4G) and 27.24% ± 0.61% (*p* < 0.001, Figure S4G), and this was increased unexpectedly in SW480 cells (Figure S4G).

### 3.7. Artesunate Increased the Cellular Free Ca^2+^ Level

**Figure 7:**
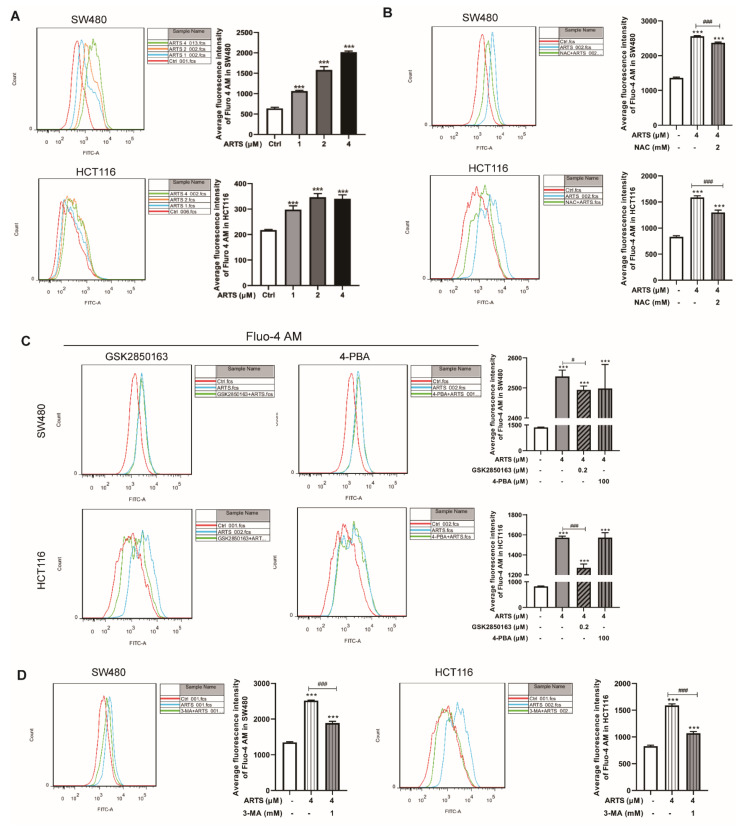
(**A**) Artesunate increased the cellular free Ca^2+^ level markedly. After artesunate treatment, cells were probed with Fluo 4-AM and analyzed by flow cytometry. The green fluorescence intensity of Fluo 4-AM represented cellular free Ca^2+^ level. (**B**) ROS scavenger NAC, (**C**) IRE1α inhibitor GSK2850163, and (**D**) autophagy inhibitor 3-MA helped to reduce cellular free Ca^2+^ level. *** *p* < 0.001 vs. Ctrl. # *p* < 0.05, ### *p* < 0.001 vs. cells treated with artesunate alone. Data were shown as mean ± SD.

Mitochondria and the endoplasmic reticulum are the main organelles that regulate the balance of cellular Ca^2+^ [31]. Due to the dysfunction caused by artesunate, Ca^2+^ is released into the cytoplasm from mitochondria and the endoplasmic reticulum. As indicated by Fluo 4-AM, artesunate increased the cellular free Ca^2+^ level in a dose-dependent manner. Compared to untreated cells, the intensity of Fluo 4-AM in SW480 cells was increased by approximately 66.70% ± 3.15% (1 μM, *p* < 0.001), 148.14% ± 12.75% (2 μM, *p* < 0.001), and 216.57% ± 4.39% (4 μM, *p* < 0.001) and was decreased by approximately 36.81% ± 7.16% (1 μM, *p* < 0.001), 59.20% ± 6.78% (2 μM, *p* < 0.001), and 56.44% ± 7.30% (4 μM, *p* < 0.001) in HCT116 cells (Figure 7A).

As shown in Figure 7B, the fluorescence intensity of Fluo 4-AM in SW480 and HCT116 cells was decreased by approximately 7.30% ± 0.36% (*p* < 0.001) and 18.10% ± 2.64% (*p* < 0.001), respectively, due to NAC exposure.

Additionally, in SW480 and HCT116 cells, GSK2850163 exposure resulted in a decrease in the fluorescence intensity of Fluo 4-AM by approximately 1.77% ± 0.51% and 19.14% ± 2.45% (*p* < 0.001), respectively. However, the fluorescence intensity of Fluo 4-AM in artesunate-treated SW480 and HCT116 cells showed no significant change after 4-PBA exposure (Figure 7C).

Notably, when cells were treated with artesunate and 3-MA simultaneously, the fluorescence intensity of Fluo 4-AM in SW480 and HCT116 cells was decreased by approximately 25.09% ± 1.92% (*p* < 0.001, Figure 7D) and 32.68% ± 1.94% (*p* < 0.001, Figure 7D), respectively.

### 3.8. Artesunate Inhibited the Growth of CT26-Derived Tumors in Vivo

**Figure 8:**
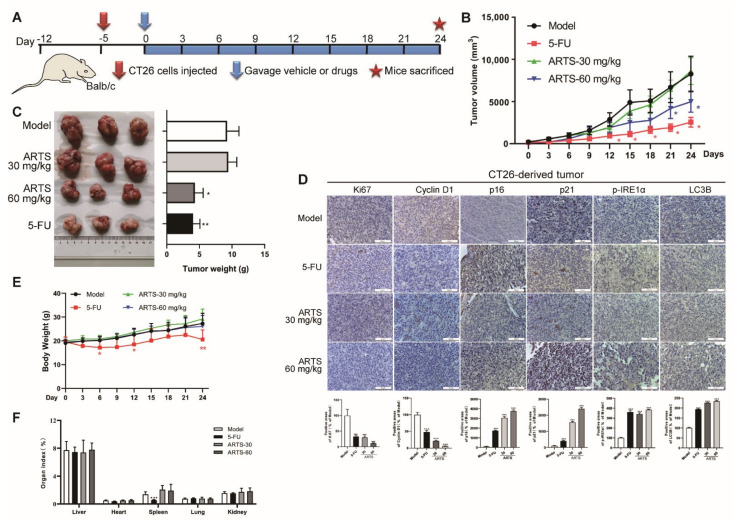
Artesunate inhibited the growth of CT26-derived tumor in vivo. (**A**) Experimental timeline of CT26-derived tumor model in balb/c mice. Balb/c mice were injected CT-26 cells (1 × 10^5^ cells for each mouse) subcutaneously to establish the CT26-derived tumor model. Tumor-loaded mice were gavaged with artesunate at 30 mg/kg or 60 mg/kg for 24 days. Body weights and tumor volumes were recorded every three days. (**B**) Curve of tumor volume. (**C**) Tumor weight. Tumor tissues were collected and weighted after treatment. (**D**) Immunohistochemical images of Ki67, Cyclin D1, p16, p21, LC3B, and p-IRE1α. Immunohistochemistry assay was performed using a SABC-POD staining kit. (**E**) Curve of body weight. (**F**) Organ indexes. Lung, heart, spleen, liver, and kidney were collected and weighted to calculate the organ indexes after treatment. * *p* < 0.05, *** *p* < 0.001 vs. Model group. Data were shown as mean ± SD.

A CT26-derived tumor model in mice was established according to the timeline shown in Figure 8A to evaluate the inhibitory effect of artesunate against colorectal cancer in vivo. Artesunate administration remarkably inhibited the growth of CT26-derived tumors, as shown by the tumor growth curves in Figure 8B and tumor weights in Figure 8C.

IHC results revealed that artesunate administration differentially reduced the expression levels of Ki67 and cyclin D1 (Figure 8D). Meanwhile, the levels of p16, p21, LC3B, and p-IRE1α were induced dramatically (Figure 8D). These findings suggested that artesunate could cause cell senescence, autophagy, and ER stress in vivo.

Furthermore, artesunate exhibited no obvious effects on body weight or organ indices, while 5-FU, a first-in-class drug for CRC therapy in clinic, caused body weight loss and repressed the spleen index (Figure 8E,F). These results indicated that artesunate at 30 mg·kg-1 and 60 mg·kg-1 was tolerable in mice without obvious toxicity.

## 4. Discussion

Artesunate is a well-known sesquiterpene lactone containing a specific peroxo bridge. Structurally, artesunate is a typical endoperoxide. Most endoperoxides, taking terpene as the basic skeleton, are reported to be potential candidates for drug discovery with good antitumor activity and antimalarial activity [32,33]. In recent years, more and more researchers are focusing on the antitumor activity of artemisinin and its derivatives and have been performing both laboratory research and clinical trials to put insight into the underlying mechanisms.

Given the specific endoperoxide function, we speculated that artesunate was likely to induce oxidative stress in cancer cells, just like arteminin and its derivatives are doing in plasmodium [8]. As expected, artesunate caused excessive mitochondrial ROS generation drastically in SW480 and HCT116 (Figure 1C). In some previous studies, artemisinin and its derivatives were reported to induce ROS-dependent cell death in some cancer cells [13,14]. However, in the present study, artesunate exhibited little effect on cell apoptosis, which has been considered to be the most common manner of cell death (Figure 2). The possible reason for these distinctive results may be related to the relatively lower concentrations (1~4 μM) we used. Hence, it is reasonable to hypothesize that some cell fates, other than apoptosis and necrosis, may be induced in an ROS-dependent manner by artesunate in our study. It is reported that artesunate could sensitize cancer cells to ferroptosis [34]. Therefore, in our study, we have applied ferrostatin-1 (a ferroptosis inhibitor) to verify whether artesunate promoted ferroptosis at 1~4 μM. However, we found that ferrostatin-1 could not help to restore the viability of SW480 and HCT116 cells (Fig.S1E). Autophagy has been reported to be a novel type of programmed cell death when overactivated [35]. In our study, we found 3-MA (an autophagy inhibitor) restored cell viability in artesunate-treated cells (Figure 5E).

Cell senescence is a permanent state of irreversible cell cycle arrest with no proliferation potential. p16 and p21 are reported markers of cellular senescence, and IL-6 and MMP3 are markers of senescence-related secretory phenotypes (SASP) [18,36]. In our study, artesunate exposure caused G1/S arrest and inhibited cell division in SW480 and HCT116 (Figure 3A and Figure S1B). Meanwhile, artesunate exposure up-regulated the protein levels of p16, p21, IL-6, and MMP3. Based on these results, we could propose that artesunate induces cell senescence in SW480 and HCT116. ROS is considered to be a leading mediator of cell senescence [37]. Through NAC, a ROS scavenger, we revealed that artesunate caused ROS-dependent cell senescence (Figure 3B and Figure S1D).

Notably, artesunate not only decreased the level of CDK2/4/6 but also increased the levels of p16 and p21, which are endogenous inhibitors of CDK2/4/6 (Figure 3C). That is to say, artesunate exhibited dual inhibitory effects on CDK2/4/6. CDK4/6 inhibitors, which induce cell senescence pharmacologically, are reported to mediate hypophosphorylation of the tumor suppressor Rb due to CDK4/6 inhibition and cause cell cycle arrest at the G1 phase [23]. In accordance, artesunate reduced the phosphorylation level of Rb (Figure S1C). However, the protein expression of Rb was repressed in artesunate-treated SW480. Meanwhile, the ratio of p-Rb/total Rb rather increased (Figure S1C). Thus, the reason for the decreased p-Rb level appeared to be due to a decrease in total Rb level, at least, in SW480. As reported, Rb is responsible for a major G1 checkpoint, blocking S-phase entry and cell growth [38]. Based on this, it seemed to be reasonable that we did not observe a reduction in the S phase population in SW480 cells.

Senescent cells are reported to be apoptosis-resistant, with Bcl-2 expression unrepressed [39]. Hence, it is reasonable that the expression of Bcl-2 was upregulated after artesunate treatment (Figure S1B), further confirming that artesunate induced cell senescence. Thus, we were convinced that artesunate inhibited cell proliferation by ROS-dependent treatment-induced senescence (TIS), which is considered to be a feasible strategy for cancer therapy [40]. Notably, the NAC effect on the viability of artesunate-treated cells is apparent (Figure 1F), but its effect on p16 levels appears to be comparatively mild in SW480 (Figure 3D). These results raised a question that senescence and autophagy occurred in a ROS-dependent manner in response to artesunate. Factually, we found that NAC repressed autophagy while reducing ROS levels (Figure 5C). Autophagy induced by artesunate was proven to inhibit cell viability in SW480 and HCT116 (Figure 5E). It is well known that senescent cells are resistant to cell death. Accordingly, we could speculate that senescence and autophagy maybe are probably two independent events that induced by artesunate.

As mentioned above, the changes of total Rb protein levels in SW480 and HCT116 were different when exposed to artesunate. Meanwhile, p16 levels in response to NAC also appeared to be different in two cell types. As reported, SW480 is a microsatellite stable (MSS) cell line with a nonsense mutation in p53, whereas HCT116 is a microsatellite instable (MSI) cell line expressing wild-type p53 [41]. Microsatellite status has been proven to affect the response to chemotherapy [42]. Given that SW480 (with a nonsense mutation in p53) and HCT116 (with wild-type p53) are both sensitive to artesunate and respond to artesunate similarly at most aspects, we speculated that microsatellite status should play a more important role for SW480 and HCT116 in exhibiting some different responses to artesunate and NAC at p16 expression.

Due to the senescence-associated secretory phenotype (SASP) in senescent cells, activated synthesis behaviors of secretory proteins in the ER probably lead to the accumulation of secretory proteins, which cause ER stress and initiate UPR [18]. Resultantly, BIP, the ER chaperone with good affinity towards unfolded and misfolded proteins, was largely increased at the translational level in artesunate-treated cells (Figure 6A). IRE1α signaling, a branch of the UPR pathways, was activated drastically (Figure 6A). Generally, the IRE1α pathway mediates cell survival via XBP1S-inhibited gene transcription [43,44], but mediates cell death mainly due to JNK activation [45]. We observed upregulation of XBP1S (Figure S4A). However, JNK1/2 was not activated after artesunate exposure (Figure S4A), which may be explained by the notion that p21 prevents cell death by restricting JNK and caspase signaling in senescent cells [46]. Notably, MMP3 and IL-6, two well-known senescence markers [18], were upregulated after artesunate treatment in vivo (Figure S4H). These findings indicated that IRE1α activation observed in our study may be just an adaptive response to the SASP of senescent cells induced by artesunate, seemingly making little sense in cell death or cell survival. PERK pathway, another branch of UPR, involves in senescence [47]. CHOP is a proven target of ATF4 in PERK pathway and is proven to be a transcription factor of DR5 [48]. Both CHOP and DR5 were upregulated remarkably in artesunate-treated cells (Figure 6A). Activated CHOP could transcriptionally induce Atg5 [49]. Autophagy-dependent DR5 upregulation has been reported to be related to cell death [50]. In our study, artesunate was proven to activate autophagy to inhibit cell viability in SW480 and HCT116. Thus, CHOP and DR5 upregulation in artesunate-treated SW480 and HCT116 may be involved in the activation of autophagy.

Mitochondria are the main cellular sources of ROS. In our study, using an intracellular ROS indicator and a mitochondrial superoxide indicator, we found that artesunate caused mitochondrial ROS generation (Figure 1C). Furthermore, we observed that artesunate caused mitochondrial depolarization (Figure S2B) prior to excessive mitochondrial ROS generation (Figure S2C). With the accumulation of dysfunctional mitochondria, mitophagy was activated evidently (Figure 4B), which could not be attenuated by the ROS scavenger NAC (Figure S2A). Thus, we were convinced that in our study, artesunate-mediated ROS generation was promoted by mitochondrial dysfunction.

ROS and IRE1α have been reported to induce autophagy [51,52]. Thus, we speculated that artesunate could activate autophagy. In present study, we found that NAC, 4-PBA, and GSK2850163 could attenuate LC3B expression (Figure 5F and Figure S4F), suggesting that artesunate may activate autophagy depending on ROS and ER stress. As mentioned previously, ER stress and UPR in artesunate-treated cells were more likely a response to the SASP of ROS-induced cell senescence. Hence, we asserted that artesunate activated autophagy in an ROS-dependent manner. Numerous studies have indicated that autophagy is a novel type of programmed cell death when overactivated [35]. Surprisingly, further study revealed that activated autophagy in artesunate-treated cells played a role in inhibiting cell viability, as confirmed by using the autophagy inhibitor 3-MA, suggesting that artesunate overactivated autophagy to cause cell death in CRC cells (Figure 5E). Inhibited autophagy may block the removal of dysfunctional mitochondria and result in ROS accumulation [53,54]. Consistently, in our study, 3-MA not only restored cell viability but also helped to elevate ROS levels (Figure S3E). Thus, ROS-induced autophagy helped to attenuate ROS generation in artesunate-treated colorectal cancer cells, namely, a negative feedback loop was formed between activated autophagy and induced mitochondrial ROS. Fortunately, ROS levels were high enough that the ROS-dependent inhibitory effect of artesunate on CRC cells was not offset by autophagy-induced negative feedback.

## 5. Conclusions

In conclusion, we have demonstrated that artesunate functions as a powerful senescence and autophagy inducer to inhibit CRC growth by targeting mitochondria and causing excessive mitochondrial ROS generation. Our data revealed that (I) artesunate targeted mitochondria to mediate mitochondrial dysfunction and resulted in mitochondrial ROS production; (II) due to excessive mitochondrial ROS, artesunate inhibited CRC growth by inducing p16- and p21-dependent cell senescence and caused cell cycle arrest but not by promoting cell apoptosis; (III) artesunate overactivated autophagy in an ROS-dependent manner to inhibit cell viability; and (IV) artesunate activated IRE1α signaling to cope with senescence-related ER stress. Our study describes a mechanism by which artesunate mediates the complex network of mitochondrial ROS, cell senescence, ER stress, and autophagy to inhibit CRC growth. Our findings suggest that inducing ROS-dependent senescence could be an effective strategy to exert the anticancer activity of artesunate.

## Data Availability

Not applicable.

## Supplementary Materials

The following supporting information can be downloaded at: https://www.mdpi.com/article/10.3390/cells11162472/s1, Figure S1: Artesunate caused cell senescence; Figure S2: Artesunate induced mitophagy due to mitochondria depolarization; Figure S3: Artesunate induced autophagy; Figure S4: Artesunate activated IRE1α signaling in UPR.

## References

[1] Sung H., Ferlay J., Siegel R.L., Laversanne M., Soerjomataram I., Jemal A., Bray F. (2021). Global Cancer Statistics 2020: GLO-BOCAN Estimates of Incidence and Mortality Worldwide for 36 Cancers in 185 Countries. CA Cancer J. Clin..

[2] Xi Y., Xu P. (2021). Global colorectal cancer burden in 2020 and projections to 2040. Transl. Oncol..

[3] Benson A.B., Venook A.P., Al-Hawary M.M., Arain M.A., Chen Y.-J., Ciombor K.K., Cohen S., Cooper H.S., Deming D., Farkas L. (2021). Colon Cancer, Version 2.2021, NCCN Clinical Practice Guidelines in Oncology. J. Natl. Compr. Cancer Netw..

[4] Li J., Casteels T., Frogne T., Ingvorsen C., Honoré C., Courtney M., Huber K.V.M., Schmitner N., Kimmel R.A., Romanov R.A. (2017). Artemisinins Target GABA(A) Receptor Signaling and Impair α Cell Identity. Cell.

[5] Fan M., Li Y., Yao C., Liu X., Liu J., Yu B. (2018). DC32, a Dihydroartemisinin Derivative, Ameliorates Collagen-Induced Arthritis Through an Nrf2-p62-Keap1 Feedback Loop. Front. Immunol..

[6] Efferth T. (2017). From ancient herb to modern drug: Artemisia annua and artemisinin for cancer therapy. Semin. Cancer Biol..

[7] Krishna S., Ganapathi S., Ster I.C., Saeed M.E., Cowan M., Finlayson C., Kovacsevics H., Jansen H., Kremsner P.G., Efferth T. (2014). A Randomised, Double Blind, Placebo-Controlled Pilot Study of Oral Artesunate Therapy for Colorectal Cancer. eBioMedicine.

[8] Gopalakrishnan A.M., Kumar N. (2015). Antimalarial Action of Artesunate Involves DNA Damage Mediated by Reactive Oxygen Species. Antimicrob. Agents Chemother..

[9] Wilson C., Muñoz-Palma E., González-Billault C. (2018). From birth to death: A role for reactive oxygen species in neuronal de-velopment. Semin. Cell Dev. Biol..

[10] Ghosh N., Das A., Chaffee S., Roy S., Sen C.K., Chatterjee S., Jungraithmayr W., Bagchi D. (2018). Reactive oxygen species, oxidative damage and cell death. Immunity and Inflammation in Health and Disease.

[11] Schmidt H.H., Ghezzi P., Cuadrado A. (2021). Reactive Oxygen Species: Network Pharmacology and Therapeutic Applications.

[12] Zou Z., Chang H., Li H., Wang S. (2017). Induction of Reactive Oxygen Species: An Emerging Approach for Cancer Therapy. Apoptosis.

[13] Gao W., Xiao F., Wang X., Chen T. (2013). Artemisinin induces A549 cell apoptosis dominantly via a reactive oxygen spe-cies-mediated amplification activation loop among caspase-9, -8 and -3. Apoptosis.

[14] Zhang Q., Yi H., Yao H., Lu L., He G., Wu M., Zheng C., Li Y., Chen S., Li L. (2021). Artemisinin Derivatives Inhibit Non-small Cell Lung Cancer Cells Through Induction of ROS-dependent Apoptosis/Ferroptosis. J. Cancer.

[15] Li S., Chaudhary S.C., Zhao X., Gaur U., Fang J., Yan F., Zheng W. (2019). Artemisinin Protects Human Retinal Pigmented Epi-thelial Cells Against Hydrogen Peroxide-induced Oxidative Damage by Enhancing the Activation of AMP-active Protein Ki-nase. Int. J. Biol. Sci..

[16] Pushpakom S., Iorio F., Eyers P.A., Escott K.J., Hopper S., Wells A., Doig A., Guilliams T., Latimer J., Mcnamee C. (2019). Drug repurposing: Progress, challenges and recommendations. Nat. Rev. Drug Discov..

[17] Dan Dunn J., Alvarez L.A., Zhang X., Soldati T. (2015). Reactive oxygen species and mitochondria: A nexus of cellular homeostasis. Redox Biol..

[18] Hernandez-Segura A., Nehme J., Demaria M. (2018). Hallmarks of Cellular Senescence. Trends Cell Biol..

[19] Lee B.Y., Han J.A., Im J.S., Morrone A., Johung K., Goodwin E.C., Kleijer W.J., Dimaio D., Hwang E.S. (2006). Senes-cence-associated beta-galactosidase is lysosomal beta-galactosidase. Aging Cell.

[20] Banks H.T., Sutton K.L., Thompson C., Bocharov G., Roose D., Schenkel T., Meyerhans A. (2010). Estimation of Cell Proliferation Dynamics Using CFSE Data. Bull. Math. Biol..

[21] Sharpless N.E., Sherr C.J. (2015). Forging a signature of in vivo senescence. Nat. Rev. Cancer.

[22] Wiley C.D., Flynn J.M., Morrissey C., Lebofsky R., Shuga J., Dong X., Unger M.A., Vijg J., Melov S., Campisi J. (2017). Analysis of individual cells identifies cell-to-cell variability following induction of cellular senescence. Aging Cell.

[23] Miettinen T.P., Peltier J., Härtlova A., Gierliński M., Jansen V.M., Trost M., Björklund M. (2018). Thermal proteome profiling of breast cancer cells reveals proteasomal activation by CDK4/6 inhibitor palbociclib. EMBO J..

[24] Gallage S., Gil J. (2016). Mitochondrial Dysfunction Meets Senescence. Trends Biochem. Sci..

[25] Ma K., Chen G., Li W., Kepp O., Zhu Y., Chen Q. (2020). Mitophagy, Mitochondrial Homeostasis, and Cell Fate. Front. Cell Dev. Biol..

[26] Green D.R., Levine B. (2014). To Be or Not to Be? How Selective Autophagy and Cell Death Govern Cell Fate. Cell.

[27] Pankiv S., Clausen T.H., Lamark T., Brech A., Bruun J.A., Outzen H., Øvervatn A., Bjørkøy G., Johansen T. (2007). p62/SQSTM1 binds directly to Atg8/LC3 to facilitate degradation of ubiquitinated protein aggregates by autophagy. J. Biol. Chem..

[28] Cao S.S., Kaufman R.J. (2014). Endoplasmic Reticulum Stress and Oxidative Stress in Cell Fate Decision and Human Disease. Antioxid. Redox Signal..

[29] Pluquet O., Pourtier A., Abbadie C. (2015). The unfolded protein response and cellular senescence. A Review in the Theme: Cellular Mechanisms of Endoplasmic Reticulum Stress Signaling in Health and Disease. Am. J. Physiol. Cell Physiol..

[30] Gardner B.M., Pincus D., Gotthardt K., Gallagher C.M., Walter P. (2013). Endoplasmic Reticulum Stress Sensing in the Unfolded Protein Response. Cold Spring Harb. Perspect. Biol..

[31] Marchi S., Patergnani S., Missiroli S., Morciano G., Rimessi A., Wieckowski M.R., Giorgi C., Pinton P. (2017). Mitochondrial and endoplasmic reticulum calcium homeostasis and cell death. Cell Calcium.

[32] Dembitsky V.M., Ermolenko E., Savidov N., Gloriozova T.A., Proroikov V.V. (2021). Antiprotozoal and Antitumor Activity of Natural Polycyclic Endoperoxides: Origin, Structures and Biological Activity. Molecules.

[33] Bu M., Yang B.B., Hu L. (2016). Natural Endoperoxides as Drug Lead Compounds. Curr. Med. Chem..

[34] Chen G.-Q., Benthani F.A., Wu J., Liang D., Bian Z.-X., Jiang X. (2019). Artemisinin compounds sensitize cancer cells to ferroptosis by regulating iron homeostasis. Cell Death Differ..

[35] Fulda S., Kögel D. (2015). Cell death by autophagy: Emerging molecular mechanisms and implications for cancer therapy. Oncogene.

[36] Davalli P., Mitic T., Caporali A., Lauriola A., D’Arca D. (2016). ROS, Cell Senescence, and Novel Molecular Mechanisms in Aging and Age-Related Diseases. Oxidative Med. Cell. Longev..

[37] Graceffa V. (2020). Therapeutic Potential of Reactive Oxygen Species: State of the Art and Recent Advances. SLAS Technol. Transl. Life Sci. Innov..

[38] Giacinti C., Giordano A. (2006). RB and cell cycle progression. Oncogene.

[39] Ryu S.J., Oh Y.S., Park S.C. (2007). Failure of stress-induced downregulation of Bcl-2 contributes to apoptosis resistance in senescent human diploid fibroblasts. Cell Death Differ..

[40] Lee S., Lee J.S. (2019). Cellular senescence: A promising strategy for cancer therapy. BMB Rep..

[41] Ahmed D., Eide P.W., Eilertsen I.A., Danielsen S.A., Eknaes M., Hektoen M., Lind G.E., Lothe R.A. (2013). Epigenetic and genetic features of 24 colon cancer cell lines. Oncogenesis.

[42] Gupta R., Sinha S., Paul R.N. (2018). The impact of microsatellite stability status in colorectal cancer. Curr. Probl. Cancer.

[43] Gonnella R., Montani M.G., Guttieri L., Romeo M., Santarelli R., Cirone M. (2021). IRE1 Alpha/XBP1 Axis Sustains Primary Effusion Lymphoma Cell Survival by Promoting Cytokine Release and STAT3 Activation. Biomedicines.

[44] McCarthy N., Dolgikh N., Logue S., Patterson J.B., Zeng Q., Gorman A.M., Samali A., Fulda S. (2020). The IRE1 and PERK arms of the unfolded protein response promote survival of rhabdomyosarcoma cells. Cancer Lett..

[45] Kato H., Nakajima S., Saito Y., Takahashi S., Katoh R., Kitamura M. (2011). mTORC1 serves ER stress-triggered apoptosis via selective activation of the IRE1–JNK pathway. Cell Death Differ..

[46] Yosef R., Pilpel N., Papismadov N., Gal H., Ovadya Y., Vadai E., Miller S., Porat Z., Ben-Dor S., Krizhanovsky V. (2017). p21 maintains senescent cell viability under persistent DNA damage response by restraining JNK and caspase signaling. EMBO J..

[47] Horiguchi M., Koyanagi S., Okamoto A., Suzuki S.O., Matsunaga N., Ohdo S. (2012). Stress-Regulated Transcription Factor ATF4 Promotes Neoplastic Transformation by Suppressing Expression of the INK4a/ARF Cell Senescence Factors. Cancer Res..

[48] Chen P., Hu T., Liang Y., Li P., Chen X., Zhang J., Ma Y., Hao Q., Wang J., Zhang P. (2016). Neddylation Inhibition Ac-tivates the Extrinsic Apoptosis Pathway through ATF4-CHOP-DR5 Axis in Human Esophageal Cancer Cells. Clin. Cancer Res..

[49] Sano R., Reed J.C. (2013). ER stress-induced cell death mechanisms. Biochim. Biophys. Acta (BBA)-Mol. Cell Res..

[50] Chen L., Meng Y., Sun Q., Zhang Z., Guo X., Sheng X., Tai G., Cheng H., Zhou Y. (2016). Ginsenoside compound K sensitizes human colon cancer cells to TRAIL-induced apoptosis via autophagy-dependent and -independent DR5 upregulation. Cell Death Dis..

[51] Poillet-Perez L., Despouy G., Delage-Mourroux R., Boyer-Guittaut M. (2014). Interplay between ROS and autophagy in cancer cells, from tumor initiation to cancer therapy. Redox Biol..

[52] Yan C., Liu J., Gao J., Sun Y., Zhang L., Song H., Xue L., Zhan L., Gao G., Ke Z. (2019). IRE1 promotes neurodegeneration through autophagy-dependent neuron death in the Drosophila model of Parkinson’s disease. Cell Death Dis..

[53] Zhan Y., Wang K., Li Q., Zou Y., Chen B., Gong Q., Ho H.I., Yin T., Zhang F., Lu Y. (2018). The Novel Autophagy Inhibitor Alpha-Hederin Promoted Paclitaxel Cytotoxicity by Increasing Reactive Oxygen Species Accumulation in Non-Small Cell Lung Cancer Cells. Int. J. Mol. Sci..

[54] Kaminskyy V., Piskunova T., Zborovskaya I.B., Tchevkina E.M., Zhivotovsky B. (2012). Suppression of basal autophagy reduces lung cancer cell proliferation and enhances caspase-dependent and -independent apoptosis by stimulating ROS formation. Autophagy.

