# Supplementary material for: Artesunate Inhibits the Cell Growth in Colorectal Cancer by Promoting ROS-Dependent Cell Senescence and Autophagy
Source: Cells. 2022 Aug 9;11(16):2472. doi: 10.3390/cells11162472 (PMC9406496; doi:10.3390/cells11162472)
Supplement: Supplementary file 1 [file cells-11-02472-s001.zip › cells-1761989-supplementary.pdf]

Supplementary Figures

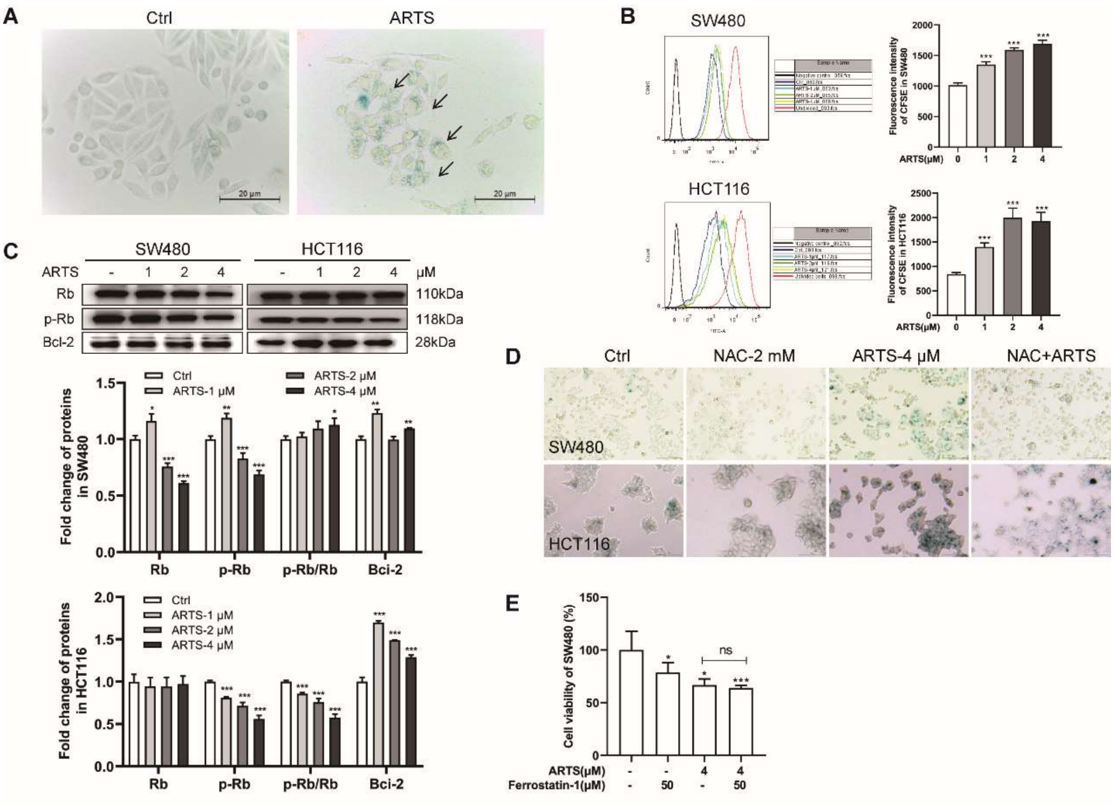

Figure S1. Artesunate caused cell senescence ROS-dependently. (**A**) Senescent cells exhibited enlarged and more flattened morphology. (**B**) Artesunate inhibited cell division in SW480 and HCT116. Cells were seeded in 6-well plates and treated with artesunate for 72 h. After treatment, cells were incubated with CFSE at a final concentration of 5 μM for 30 min and cultured in CFSE-free medium (containing 10% FBS) for 6 hours more before flow cytometry. (**C**) Artesunate repressed the expression levels of Rb, p-Rb. The expression of Bcl-2 was unrepressed. The gray values of protein blots were evaluated by Image J. Relative protein expression was normalized to β-actin in Figure 3. (**D**) Artesunate-induced cell senescence could be attenuated by NAC. NAC was used at a concentration of 2 mM and added alone or together with artesunate (4 μM) for 72 h. (**E**) Ferrostatin-1 could not help to restore cell viability. Cells were seeded in 6-well plates and treated with for 72 h. Ferrostatin-1 was used at a concentration of 50 μM and added alone or together with artesunate (4 μM). Cell viability was determined using CCK8 assay. *$p < 0.05$, **$p < 0.01$, and ***$p < 0.001$ vs. Ctrl. Data were shown as mean ± SD.

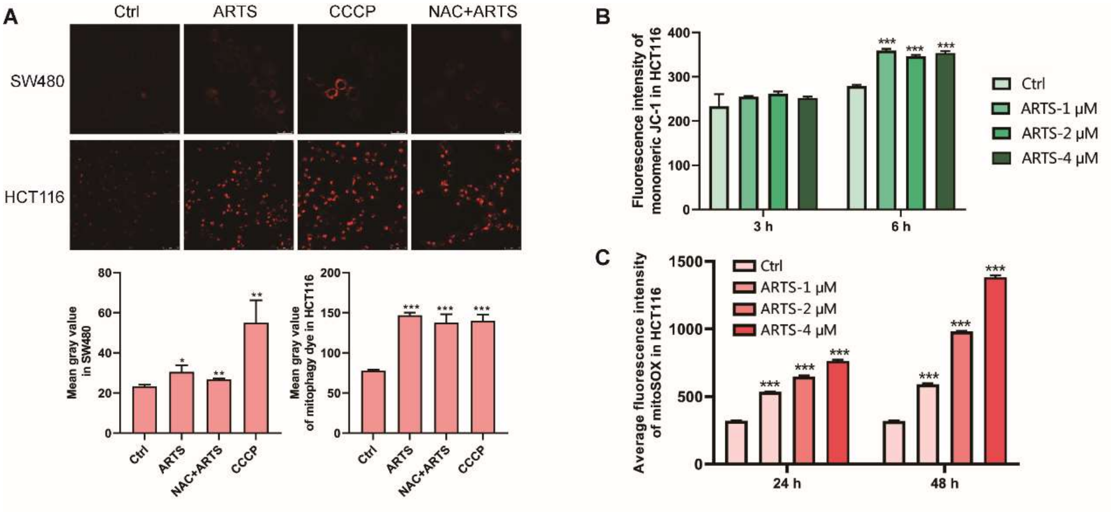

Figure S2. Artesunate induced mitophagy due to mitochondria depolarization**.** (**A**) Artesunate induced mitophagy due to mitochondria depolarization, which was indicated by a positive drug CCCP. Artesunate caused (**B**) mitochondria depolarization prior to (**C**) excessive ROS generation. *$p < 0.05$, **$p < 0.01$, and ***$p < 0.001$ vs. Ctrl. Data were shown as mean ± SD.

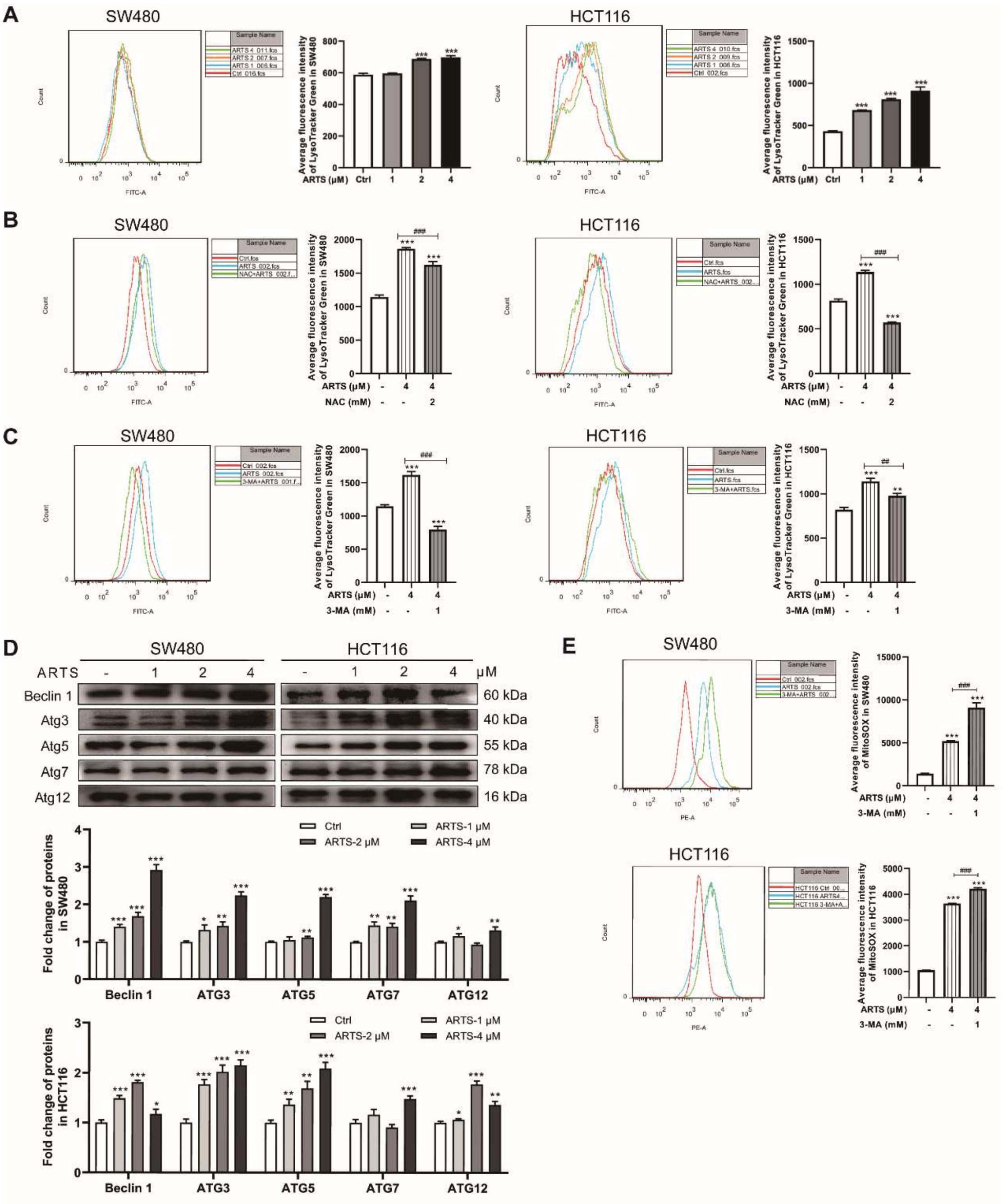

Figure S3. Artesunate induced autophagy ROS-dependently. (**A**) Artesunate promoted lysosomal acidification. Cells were probed with LysoTracker® Green DND-26 after artesunate treatment. The fluorescence intensity of LysoTracker® Green DND-26 was analyzed by flow cytometry to indicate the lysosomal acidification level. (**B**) NAC and (**C**) 3-MA helped to reduce the fluorescence intensity of LysoTracker® Green DND-26. The fluorescence intensity of LysoTracker® Green DND-26 was evaluated by flow cytometry. (**D**) The expression levels of Beclin 1, Atg 3, Atg 5, Atg 7, Atg 12 were analyzed by western blotting. The gray values of protein blots were evaluated by Image J. Relative protein expression was normalized to β-actin in Figure 5. (**E**) 3-MA helped to elevate ROS levels. Cells were probed with MitoSOX™ Red after artesunate treatment. The fluorescence intensity of MitoSOX™ Red was analyzed by flow cytometry. *$p < 0.05$, **$p < 0.01$, and ***$p < 0.001$ vs. Ctrl. ##$p < 0.01$, and ###$p < 0.001$ vs. cells treated with artesunate alone. Data were shown as mean ± SD.

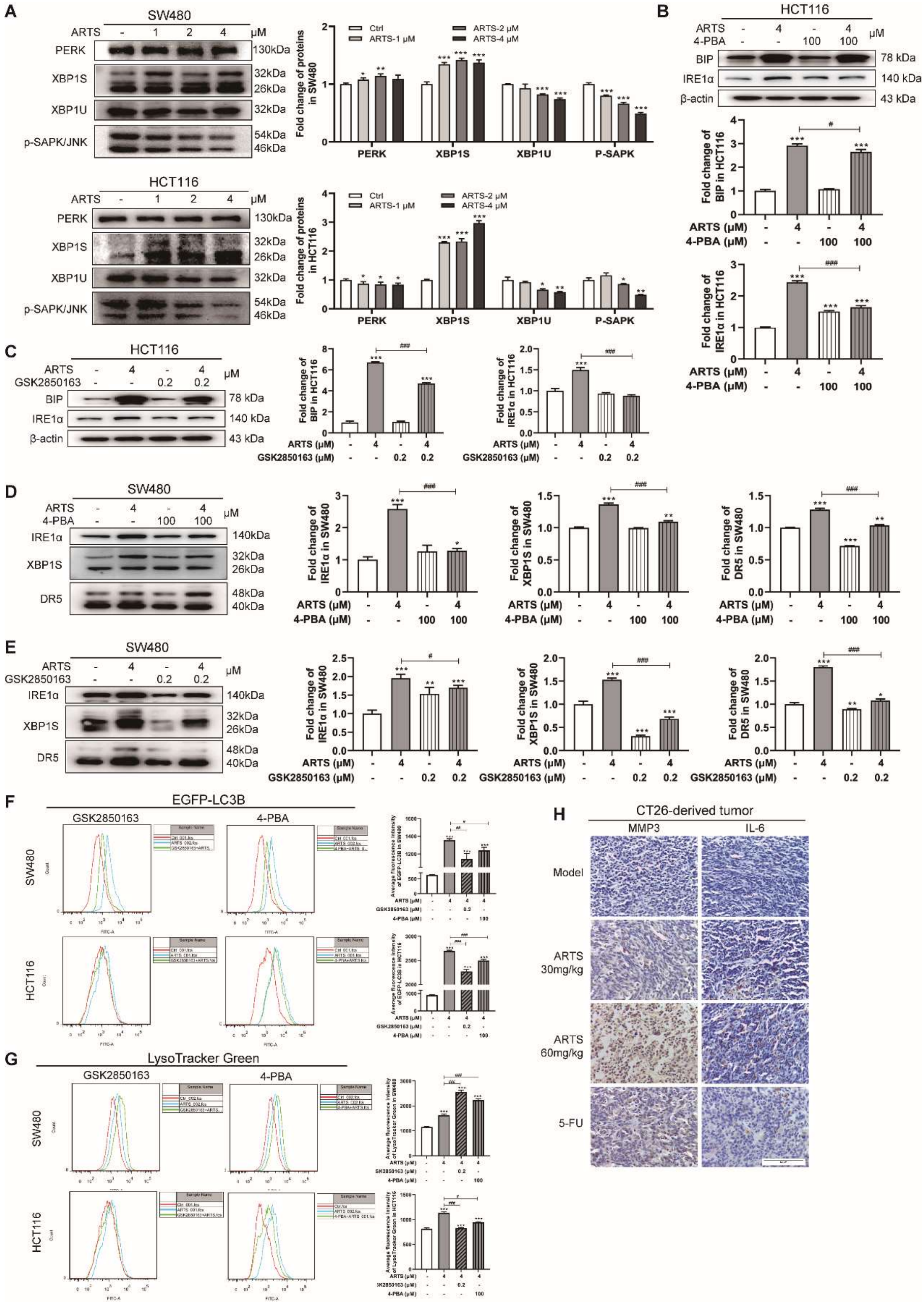

Figure S4. Aresunate activated IRE1α signaling in UPR. (**A**) The expression levels of PERK, XBP1S, XBP1U and p-SAPK/JNK were measured by western blotting. The gray values of protein blots were evaluated by Image J. Relative protein expression was normalized to β-actin in Figure 6A. (**B**) 4-PBA and (**C**) GSK2850163 helped to repress the expression level of BIP and IRE1α in HCT116. (**D** & **E**) The expression level of IRE1α, XBP1S and DR5 were measured by western blotting. The gray values of protein blots were evaluated by Image J. Relative protein expression was normalized to β-actin. (**F**) 4-PBA and GSK2850163 helped to reduce the fluorescence intensity of EGFP-LC3B in

SW480 and HCT116. The gray values of protein blots were evaluated by Image J. Relative protein expression was normalized to β-actin in Figure 6B or Figure 6C. (**G**) 4-PBA and GSK2850163 only helped to reduce the fluorescence intensity of LysoTracker® Green DND-26 in HCT116. (**H**) Artesunate promoted MMP3 and IL-6 protein expression *in vivo*. *$p < 0.05$, **$p < 0.01$, and ***$p < 0.001$ vs. Ctrl. #$p < 0.05$, ##$p < 0.01$, and ###$p < 0.001$ vs. cells treated with artesunate alone. Data were shown as mean ± SD.
